# KDM4A regulates microglial polarization after ischemic stroke by regulating SPINK5 signaling: Role of KDM4A in ischemic stroke

**DOI:** 10.3724/abbs.2025132

**Published:** 2025-09-26

**Authors:** Xiaoli Min, Lei Xian, Ting Liu, Mengze Wang, Qing Zhao, Jiayi Hu, Rui Jing

**Affiliations:** 1 Department of Cerebrovascular Diseases the Second Affiliated Hospital of Kunming Medical University Kunming 650101 China; 2 Department of Neurology the Fourth Affiliated Hospital of Xinjiang Medical University Xinjing 830054 China; 3 Department of Neurosurgery Affiliated Children’s Hospital of Kunming Medical University Kunming 650103 China

**Keywords:** ischemic stroke, microglial polarization, KDM4A, SPINK5, apoptosis

## Abstract

Microglia/macrophage polarization is a crucial factor in inflammatory processes following ischemic stroke (IS). This study explores the molecular mechanisms through which lysine-specific histone demethylase 4 (KDM4A) regulates microglial polarization postischemic stroke. IS models are established *in vivo* via transient middle cerebral artery occlusion (MCAO) surgery and *in vitro* via oxygen-glucose deprivation (OGD) treatment. 2,3,5-Triphenyl tetrazolium chloride staining is conducted to determine the infarct size. RT-qPCR is used to determine mRNA expression. Immunofluorescence assay is used to detect the expressions of KDM4A and biomarkers of microglia. Western blot analysis is used to determine the expressions of KDM4A and serine peptidase inhibitor Kazal type 5 (SPINK5). The enrichment of H3K9me3 on the promoter of *SPINK5* is determined via chromatin immunoprecipitation assay. Neuronal apoptosis is detected via TUNEL assay. We find that KDM4A is upregulated in IS models. Downregulation of KDM4A mitigates neurological dysfunction, enhances motor capacity, and reduces inflammatory infiltration *in vivo* while suppressing microglial activation and promoting M2 polarization. Mechanistically, KDM4A reduces the enrichment of H3K9me3 on the *SPINK5* promoter, thereby increasing SPINK5 expression. Moreover, overexpression of SPINK5 inhibits M2 microglial polarization and neuronal apoptosis. Overall, KDM4A exacerbates IS-induced brain injury by promoting proinflammatory microglial polarization via SPINK5 signaling.

## Introduction

Stroke is the leading cause of death and disability globally [1]. Ischemic stroke (IS), accounting for nearly 85% of all stroke cases [2], is induced by a myriad of factors, including cerebral hypoperfusion, genetic predispositions, and the environment [ 3 , 4]. Despite significant advancements in treatment modalities, the incidence of IS continues to increase annually, with clinical outcomes remaining suboptimal [ 5, 6]. Therefore, unveiling the underlying mechanisms is crucial for developing novel therapeutic strategies for IS.

The cytokine‐dependent microenvironment is the key factor that contributes to the progression of IS [7]. The inflammatory response is a crucial driver of secondary injury after stroke [8]. IS injury activates macrophages/microglia, promoting their infiltration into stroke lesions [9]. Under various stimuli, macrophages/microglia can switch to the M1 phenotype, releasing proinflammatory cytokines ( *e.g*., IL-6 and IL-1β) that induce neuronal inflammation and damage or M2 polarization, which promotes tissue repair through anti-inflammatory cytokines ( *e.g*., IL-10) [ 10– 14]. Thus, investigating microglial plasticity holds promise for IS treatment.

Lysine-specific demethylase 4A (KDM4A, or JMJD2A), a member of the KDM4 histone demethylase family [15], erases H3K9me3 and regulates diverse biological processes, such as cell proliferation, myogenesis, ferroptosis, and the immune response [ 16– 19]. Aberrant KDM4A expression is implicated in brain disorders, including IS. For example, KDM4A overexpression is associated with the carcinogenesis of breast cancer, prostate cancer, and hepatocellular carcinoma [ 20 – 22]. Targeting KDM4A inhibits inflammation and oxidative stress in hypercholesterolemia/atherosclerosis-induced heart failure [23]. Recent studies have highlighted its role in brain disorders. Huang *et al*. [24] demonstrated that KDM4A exacerbates neuroinflammation and oxidative stress. Conversely, KDM4A deficiency inhibits neuroinflammation and improves functional recovery in IS [25]. However, the comprehensive role of KDM4A in IS remains to be fully elucidated. This study investigated the roles of KDM4A in IS, hypothesizing that it may serve as a therapeutic target.

## Materials and Methods

### Specimen

Postmortem brain tissues were collected from IS patients within 1 day and 3–10 days, as well as from control subjects without neurological diseases. The tissues were stored at –80°C in lipid nitrogen. Ethical approval was granted by the Ethical Committee of the Second Affiliated Hospital of Kunming Medical University. Informed consent was provided by all patients and their guardians.

### Animals

Sprague-Dawley (SD) rats (10–12 weeks, 250–280 g; Guangzhou Ruig Biotechnology, Guangzhou, China) were housed under pathogen-free conditions at 22 ± 2°C under a 12-h light/dark cycle with free access to food and water. The animal experiments adhered to the ARRIVE guidelines. This study was approved by the Animal Care Aboard of the Second Affiliated Hospital of Kunming Medical University (KMMU20220412).

### Establishment of the IS model *in vivo*

The rats were anesthetized with 3% isoflurane and maintained with 1.5% isoflurane in 30% oxygen and 70% nitrous oxide using a face mask. The rats were randomly divided into 8 groups: sham group, MCAO group, MCAO + LV-NC group, MCAO + LV-KDM4A group, MCAO + LV-shNC group, MCAO + shKDM4A, MCAO + LV-shKDM4A + LV-NC group, and MCAO + LV-shKDM4A + LV-serine peptidase inhibitor Kazal type 5 (SPINK5) group. The right external carotid artery was exposed, and a silicone rubber-coated 6-0 nylon filament (Doccol, Sharon, USA) was inserted and advanced 9–10 mm to the carotid bifurcation along the internal carotid artery and to the origin of the middle cerebral artery (MCA). One hour after occlusion, the filament was removed to restore blood flow to the middle cerebral artery territory. In the MCAO group, LV-KDM4A, LV-shKDM4A, LV-SPINK5 or the control was delivered to tissue via Lipofectamine 3000 Transfection Reagent (Invitrogen, Carlsbad, USA) and injected into the cerebral cortex with a stereotaxic instrument after the rats were anesthetized 48 h before MCAO. The procedures performed in the sham group were the same as those in the model group, except the nylon filament. The temperature of the rats was maintained at 37.0 ± 0.5°C during the surgery and recovery periods via a temperature-controlled heating pad.

### Behavioral tests

Behavioral analysis was performed via the modified neurological severity score (mNSS), corner-turning test, foot-fault test, rotarod test, and Morris water maze test as previously described [ 26, 27].

### TTC staining

The brain tissues were collected, sliced into 1-mm-thick sections and stained with 1% TTC reagents (HY-D0714; MCE, Monmouth Junction, USA). The sections were imaged with a microscope (Nikon, Tokyo, Japan).

### Immunofluorescence assay

The slides were fixed in paraffin, deparaffinized and immersed in ethylene diamine tetraacetic acid buffer (93283, 100 mL; Sigma-Aldrich, Burlington, USA). The slides were blocked with 1% BSA and then incubated with primary antibodies, including anti-KDM4A (3393T, 1:200; CST, Boston, USA), brown adipocyte 1 (Iba1) (17198T, 1:200; CST), and CD206 (24595T, 1:1000; CST), and then with secondary antibodies (4413S, 1:200; CST). The nuclei were counterstained with 4′,6-diamidino-2-phenylindole (DAPI) (D9542; Sigma-Aldrich). Finally, images were captured via an ECLIPSE Ts2 microscope (Nikon).

### Immunohistochemistry

The brain slices were fixed in 4% paraformaldehyde for 24 h at 4°C and embedded in paraffin. After deparaffinization and blocking with 1% BSA, antigen retrieval was performed by heating the slides in a pressure cooker with 10 mM citrate buffer (pH 6.0) for 10 min. The sections were then blocked with 5% normal goat serum in phosphate-buffered saline (PBS) for 30 min at room temperature to reduce nonspecific binding. The slices were incubated with primary antibodies against KDM4A (ab191433, 1:200; Abcam, Cambridge, UK) and SPINK5 (ab246890, 1:500; Abcam) overnight at 4°C. After being washed with PBS, the sections were incubated with biotinylated secondary antibodies (ab150077, 1:500; Abcam) for 1 h at room temperature. The nuclei were counterstained with DAPI. Finally, images were captured with the ECLIPSE Ts2 microscope.

### TUNEL staining

Neuronal apoptosis in the infarct border zone was determined by terminal TUNEL staining via an *In Situ* Cell Death Detection Kit (Roche Diagnostics, Basel, Switzerland). Briefly, the brain slices were fixed in 4% paraformaldehyde for 24 h at 4°C and embedded in paraffin. Paraffin-embedded brain sections (5 μm thick) were deparaffinized in xylene and rehydrated through a graded ethanol series. Antigen retrieval was performed by heating the slides in a pressure cooker with 10 mM citrate buffer (pH 6.0) for 10 min. The sections were then blocked with 5% normal goat serum in PBS for 30 min at room temperature to reduce nonspecific binding. The sections were incubated with the TUNEL reaction mixture for 60 min at 37°C in a humidified chamber. The reaction was terminated by washing the sections with PBS. Neurons were labeled with an anti-NeuN antibody (24307S, 1:100; CST). The nuclei were counterstained with DAPI. Finally, the images were obtained via the ECLIPSE Ts2 microscope. The apoptotic index was calculated as the percentage of TUNEL-positive neurons relative to the total number of neurons.

### Cell transfection

The rat microglial cell line HAPI was provided by Yaji Biotech (Shanghai, China). The cells were cultured in Dulbecco’s modified Eagle’s medium (DMEM, 12491023; Gibco, Waltham, USA) supplemented with 10% fetal bovine serum (26010074; Gibco) in 5% CO _2_ at 37°C. The cells were stimulated with LPS and transfected with KDM4A shRNA via Lipofectamine™ 2000 (Invitrogen).

Lentivirus (LV) particles were obtained from GenePharma (Shanghai, China). KDM4A shRNA (shKDM4A#1: 5′-GCTGGCCAGTTTCTCATTTGC-3′, and shKDM4A#2: 5′-GCTTTATACTCTGTAGCTAAG-3′), shRNA-NC (5′-GCTCCCTTCAATCCAA-3′), and KDM4A/SPINK5 overexpression plasmids and the empty vector (GenePharm) were synthesized and cloned and inserted into the LV (titer for LV particles containing KDM4A-shRNA and vehicle-shRNA, KDM4A/SPINK5 overexpression plasmids and vector: 1 × 10 ^13^ vg/mL).

### Establishment of oxygen-glucose deprivation/reoxygenation

HAPI cells were maintained in glucose-free medium in a humidified incubator with 95% N _2_ and 5% CO _2_ for 2 h. Then, the medium was replaced by DMEM, and the cells were returned to a normal incubator for reoxygenation for 24 h.

### Chromatin immunoprecipitation assay

An EpiQuik Chromatin Immunoprecipitation (Co-IP) Assay kit (EpiGentek, Brooklyn, USA) was used to assess the enrichment of H3K9me3 on the *SPINK5* promoter. Briefly, the cells were crosslinked with 1% formaldehyde for 10 min at room temperature. The cross-linking reaction was quenched by adding glycine to a final concentration of 125 mM for 5 min. The cells were then washed twice with ice-cold PBS and scraped into 1 mL of PBS. The cell suspension was centrifuged at 4°C for 5 min at 1000 *g*, and the pellet was resuspended in 1 mL of lysis buffer supplemented with protease and phosphatase inhibitors. The suspension was incubated on ice for 10 min, followed by centrifugation at 4°C for 5 min at 1000 *g*. The pellet was resuspended in 1 mL of nuclear lysis buffer supplemented with protease and phosphatase inhibitors and incubated on ice for 10 min. The chromatin was sonicated to an average fragment size of 400–800 bp via a Bioruptor (Diagenode, Danville, USA) with the following settings: 30 s on, 30 s off, for 15 cycles. The sonicated chromatin was centrifuged at 4°C for 10 min at 11,200 *g*, and the supernatant was collected for the ChIP assay. The sonicated chromatin was diluted 10-fold with ChIP dilution buffer supplemented with protease and phosphatase inhibitors. The diluted chromatin was precleared with 50 μL of protein A/G magnetic beads (Millipore, Bedford, USA) for 1 h at 4°C with rotation. The precleared chromatin was then incubated with 5 μg of specific antibodies, such as an anti-H3K9me3 antibody (13969S, 1:50; CST) or a control IgG antibody (2729S, 1:50; CST), overnight at 4°C with rotation. The antibody-chromatin complexes were captured with 50 μL of protein A/G magnetic beads for 2 h at 4°C with rotation. The beads were resuspended in 100 μL of elution buffer and incubated at room temperature for 15 min with gentle agitation. The eluate was transferred to a new tube, and the beads were washed with an additional 100 μL of elution buffer. The combined eluates were incubated at 65°C for 4 h to reverse the cross-links. The samples were then treated with 0.2 mg/mL RNase A (R6513; Sigma-Aldrich) at 37°C for 30 min, followed by 0.2 mg/mL proteinase K (124568; Sigma-Aldrich) at 55°C for 1 h. The DNA was purified via the ChIP DNA Clean & Concentrator Kit (Zymo Research, Orange County, USA) according to the manufacturer’s instructions. The enrichment was subsequently detected by RT-qPCR.

### Bioinformatics analysis

Differentially expressed genes (DEGs) after KDM4A shRNA transfection were identified via the GSE63812 dataset ( https://www.aclbi.com/static/index.html#/).

### RT-qPCR

Total RNA was extracted from brain tissues via TRIzol reagent (Invitrogen) according to the manufacturer’s instructions. RNA quantity and quality were assessed via a NanoDrop spectrophotometer (Thermo Fisher Scientific, Waltham, USA) and agarose gel electrophoresis. cDNA was synthesized via SuperScript IV (11756500; Thermo Fisher Scientific) according to the manufacturer’s instructions, incubated at 42°C for 15 min, and reverse transcribed with 1 μg of total RNA at 85°C for 5 s. PCR was conducted via TaqPath™ 1-Step RT-qPCR kit (A15300; Thermo Fisher Scientific). The thermocycling conditions were as follows: 95°C for 3 min, followed by 40 cycles at 95°C for 5 s and 60°C for 30 s. *β‐Actin* served as an internal reference control. Relative mRNA levels were determined via the 2 ^‒ΔΔCq^ method. The sequences of the primers used for PCR are listed in Table 1.

**Table:** **Table 1** The sequences of the primers used in PCR

Name	Sequences (5′→3′)
*IL*- *1β*	F: GGCTTCCTTGTGCAAGTGTC R: AGTCAAGGGCTTGGAAGCAA
*TNF*- *α*	F: CCAGGTTCTCTTCAAGGGACAA R: GGTATGAAATGGCAAATCGGCT
*IL*- *6*	F: GCCCACCAGGAACGAAAGTC R: TGGCTGGAAGTCTCTTGCGG
*IL*- *10*	F: AGGGTTACTTGGGTTGCC R: GGGTCTTCAGCTTCTCTCC
*Arg1*	F: CAGTATTCACCCCGGCTA R: CCTCTGGTGTCTTCCCAA
*Ym1*	F: CTCCTCAGAACCGTCAGA R: CTCCAGTGTAGCCATCCT
*β*‐ *actin*	F: CCTAGACTTCGAGCAAGAGA R: GGAAGGAAGGCTGGAAGA

### Western blot analysis

Proteins were extracted from brain tissues via RIPA buffer (Thermo Fisher Scientific) supplemented with protease inhibitors (Roche Diagnostics). The protein concentration was determined via a bicinchoninic acid protein assay kit (Thermo Fisher Scientific). The proteins were separated by 12% sodium dodecyl sulfate-polyacrylamide gel electrophoresis and transferred to polyvinylidene fluoride membranes (IPFL00005; Millipore, Bedford, USA). The membranes were blocked with 5% skim milk. The membranes were subsequently incubated with primary antibodies, including anti-KDM4A (3393T, 1:1000; CST), SPINK5 (ab138511, 1:5000; Abcam), H3K9me3 (13969S, 1:1000; CST), H3 (9715S, 1:1000; CST), and β-actin (9715L, 1:1000; CST), overnight at 4°C, followed by incubation with goat anti-rabbit secondary antibodies (7074V, 1:3000; CST) at room temperature for 90 min. Finally, the immunoreactive bands were visualized via an enhanced chemiluminescence kit (E7750-100MG; Sigma-Aldrich).

### Statistical analysis

SPSS v.20.0 was used for data analysis. The data are expressed as the mean ± standard deviation. Intergroup comparisons were analyzed by Student’s *t* test and ANOVA. *P* < 0.05 was considered statistically significant.

## Results

### KDM4A is upregulated in the MCAO-induced IS rat model

**Figure 1:**
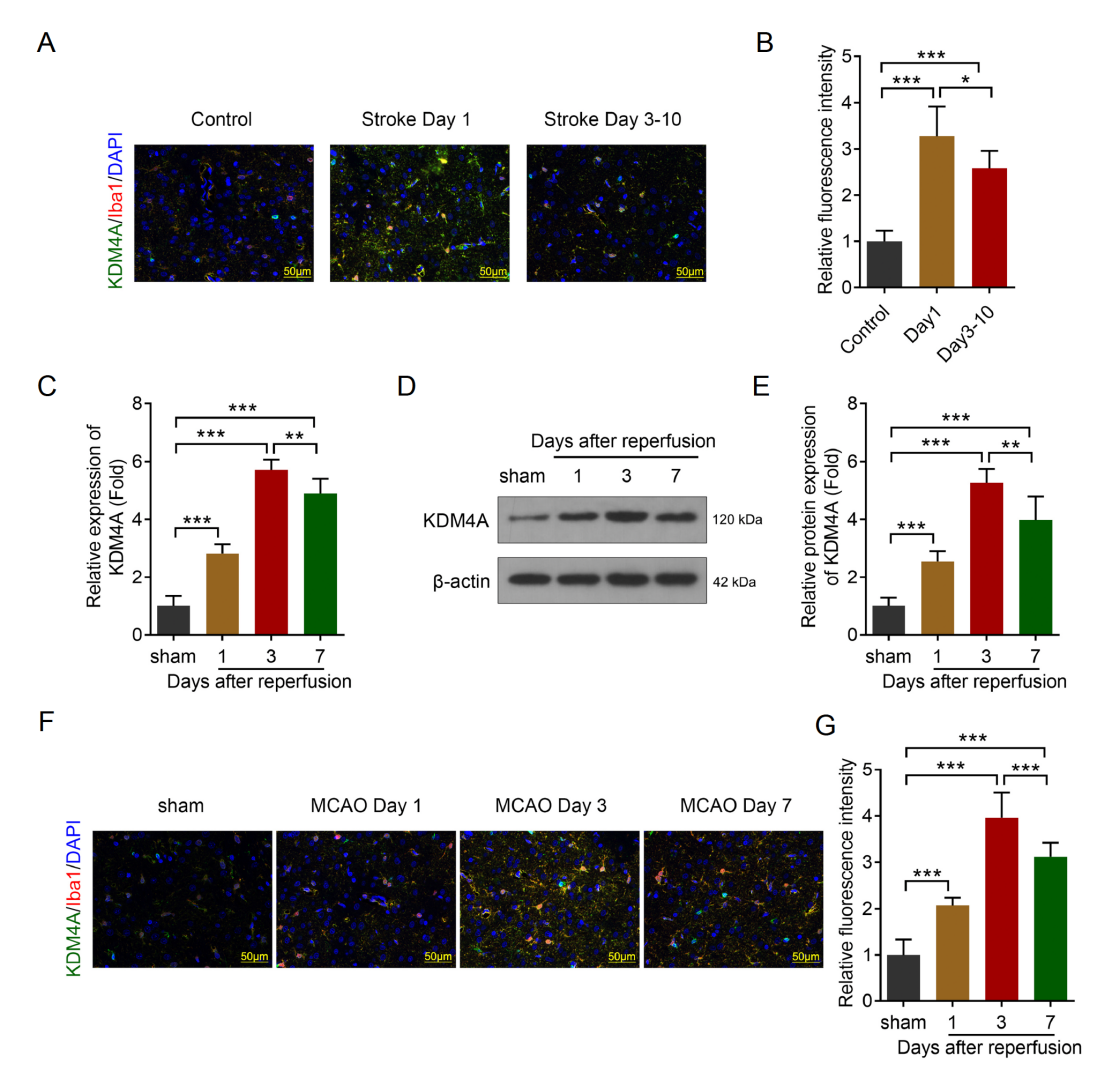
Figure 1 KDM4A is upregulated in the MCAO-induced IS rat model (A,B) KDM4A expression in the brain tissues of IS patients and healthy controls was detected via immunofluorescence assay. Scale bar: 50 μm. n = 6. (C) KDM4A mRNA expression in the brain tissues of the MCAO-induced IS model rats was detected via RT-qPCR. n = 6. (D,E) KDM4A protein expression in the brain tissues of the MCAO-induced IS model rats was detected via western blot analysis. n = 6. (F,G) KDM4A and Iba1 expression in the brain tissues of the MCAO-induced IS model rats was detected via immunofluorescence assay. Scale bar: 50 μm. n = 6. *P < 0.05, **P < 0.01, ***P < 0.001.

KDM4A overexpression contributes to the progression of IS [ 24, 25]. However, the roles of KDM5A in the microenvironment of IS have rarely been reported. KDM4A induces neuroinflammation by mediating M1 microglia polarization. From immunofluorescence assay, we found that KDM4A expression was markedly increased in IS patients ( Figure 1A,B). This finding was consistent with the results from western blot and RT-qPCR assays. The mRNA level of *KDM4A* was markedly increased in the MCAO group ( Figure 1C). KDM4A protein expression was markedly increased in the MCAO group compared with the sham group ( Figure 1D,E). Moreover, the upregulation of KDM4A was accompanied by the activation of microglia *in vivo* ( Figure 1F,G), as manifested by an increase in the percentage of KDM4A ^+^Iba1 ^+^ cells. These results suggest that the KDM4A-microglia axis may promote the progression of IS.

### Microglial KDM4A exacerbates IS-mediated brain damage

**Figure 2:**
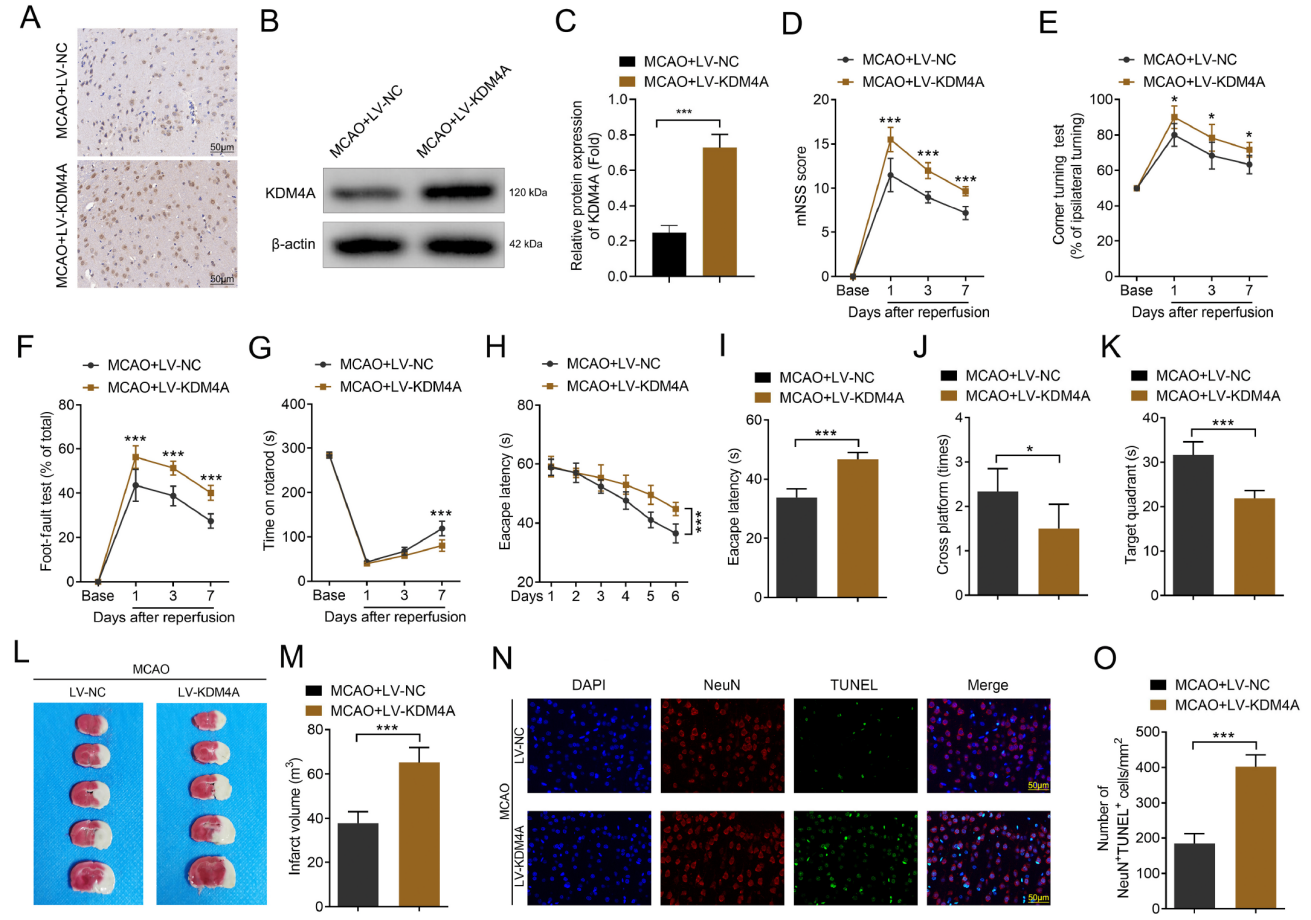
Figure 2 Microglial KDM4A exacerbates IS-mediated brain damage (A) KDM4A expression in the brain tissues of the MCAO-induced IS model rats was detected via immunohistochemistry assay. n = 6. (B,C) KDM4A protein expression in the brain tissues of the MCAO-induced IS model rats was detected via western blot analysis. n = 6. (D‒G) The neurological functions and behaviors of the MCAO-induced IS model rats were determined via the mNSS (D), corner-turning test (E), foot-fault test (F), and rotarod test (G). n = 6. (H‒K) Cognitive function was analyzed via the Morris water maze test. n = 6. (L,M) The infarct volume of brain tissues was detected via TTC staining after MCAO surgery. n = 6. (N,O) Neuronal apoptosis was determined via TUNEL assay after MCAO surgery. Scale bar: 50 μm. n = 6. *P < 0.05, ***P < 0.001.

To confirm the role of microglial KDM4A in IS, rats were injected with KDM4A overexpression plasmids after MCAO. Immunohistochemistry assay revealed that KDM4A expression was markedly increased by the overexpression plasmid ( Figure 2A). These findings paralleled the western blot analysis results. KDM4A protein expression was significantly increased by the KDM4A overexpression plasmid ( Figure 2B,C). We found that KDM4A overexpression significantly exacerbated neuroinflammation, cognitive deficits, and motor impairments ( Figure 2D–G). Compared with those in the MCAO + LV-NC group, the cognitive deficits of the rats in the MCAO + LV-KDM4A group, including decreased latency to find the submerged platform, decreased number of platform crossings and less time in the quadrant ( Figure 2H–K), were significantly greater. Moreover, TTC staining showed that KDM4A overexpression significantly increased the ischemic volume ( Figure 2L,M). Additionally, KDM4A overexpression significantly enhanced neuronal apoptosis ( Figure 2N,O). These results suggest that KDM4A may exacerbate the progression of IS.

### *KDM4A* deficiency alleviates MCAO-induced brain damage

**Figure 3:**
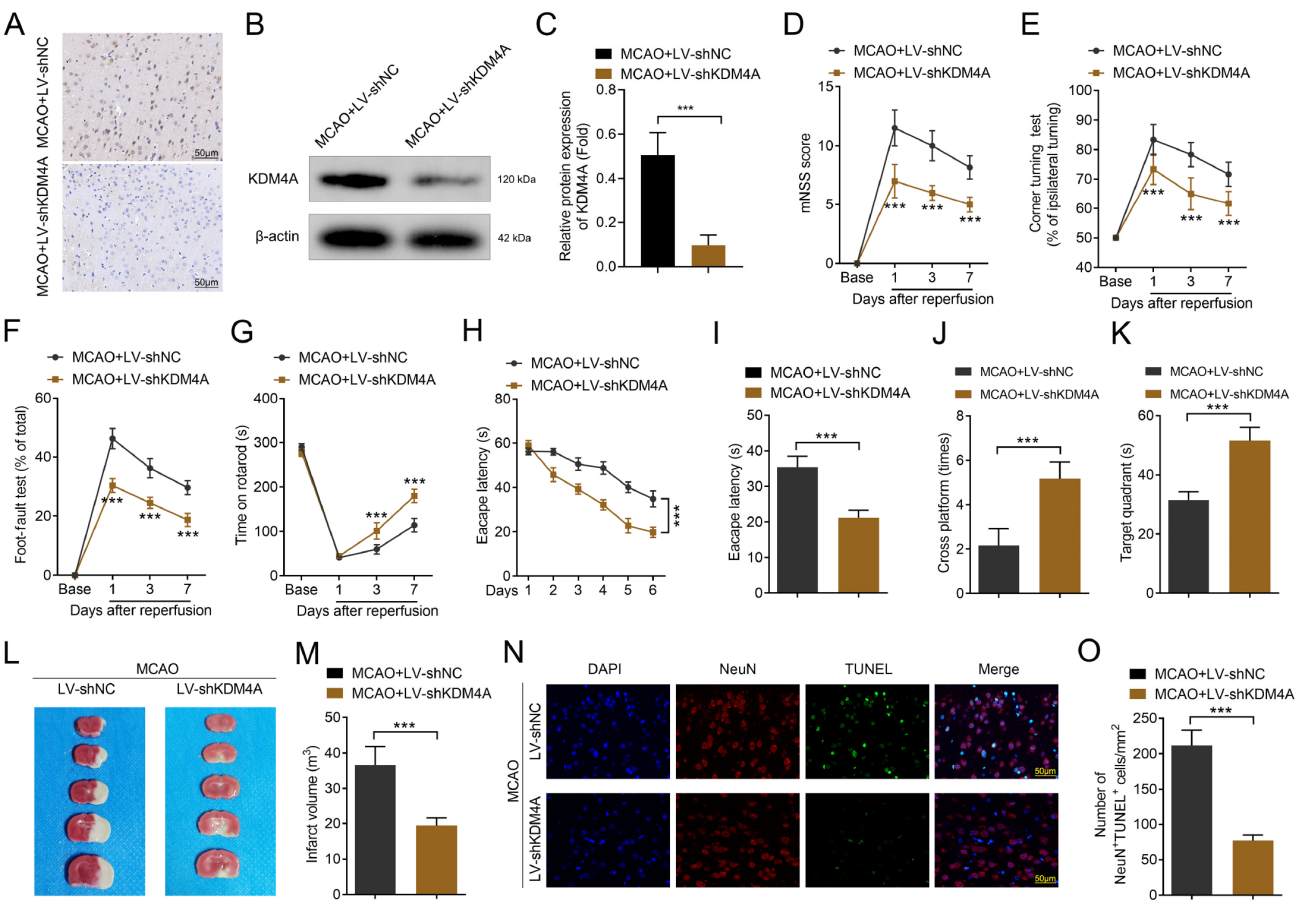
Figure 3 *KDM4A* deficiency alleviates MCAO-induced brain damage (A) KDM4A expression in the brain tissues of the MCAO-induced IS model rats was detected via immunohistochemistry assay. n = 6. (B,C) KDM4A protein expression in the brain tissues of the MCAO-induced IS model rats was detected via western blot analysis. n = 6. (D–G) The neurological functions and behaviors of the MCAO-induced IS model rats were determined via the mNSS score (D), corner-turning test (E), foot-fault test (F), and rotarod test (G). n = 6. (H‒K) Cognitive function was analyzed via the Morris water maze test. n = 6. (L,M) The infarct volume of brain tissues was detected via TTC staining after MCAO surgery. n = 6. (N,O) Neuronal apoptosis was determined via a TUNEL assay after MCAO surgery. Scale bar: 50 μm. n = 6. ***P < 0.001.

Conversely, KDM4A expression was markedly reduced by its shRNA ( Figure 3A). Moreover, KDM4A protein expression was significantly decreased by its shRNA ( Figure 3B,C), suggesting that transfection was successful. *KDM4A* knockdown in rats following MCAO surgery mitigated the adverse effects of IS. Specifically, KDM4A deficiency significantly reduced neuroinflammation, cognitive deficits, and motor impairments ( Figure 3D–G). Compared with the MCAO + LV-shNC group, the KDM4A deficiency group presented significantly restored cognitive function, including decreased latency to find the submerged platform, increased number of platform crossings and more time in the quadrant ( Figure 3H–K). Moreover, *KDM4A* knockdown significantly decreased the infarct volume ( Figure 3L,M). Additionally, *KDM4A* knockdown significantly attenuated neuronal apoptosis induced by MCAO surgery ( Figure 3N,O). These results suggested that targeting KDM4A effectively alleviated IS-induced brain damage.

### *KDM4A* deficiency inhibits M1 microglia polarization

**Figure 4:**
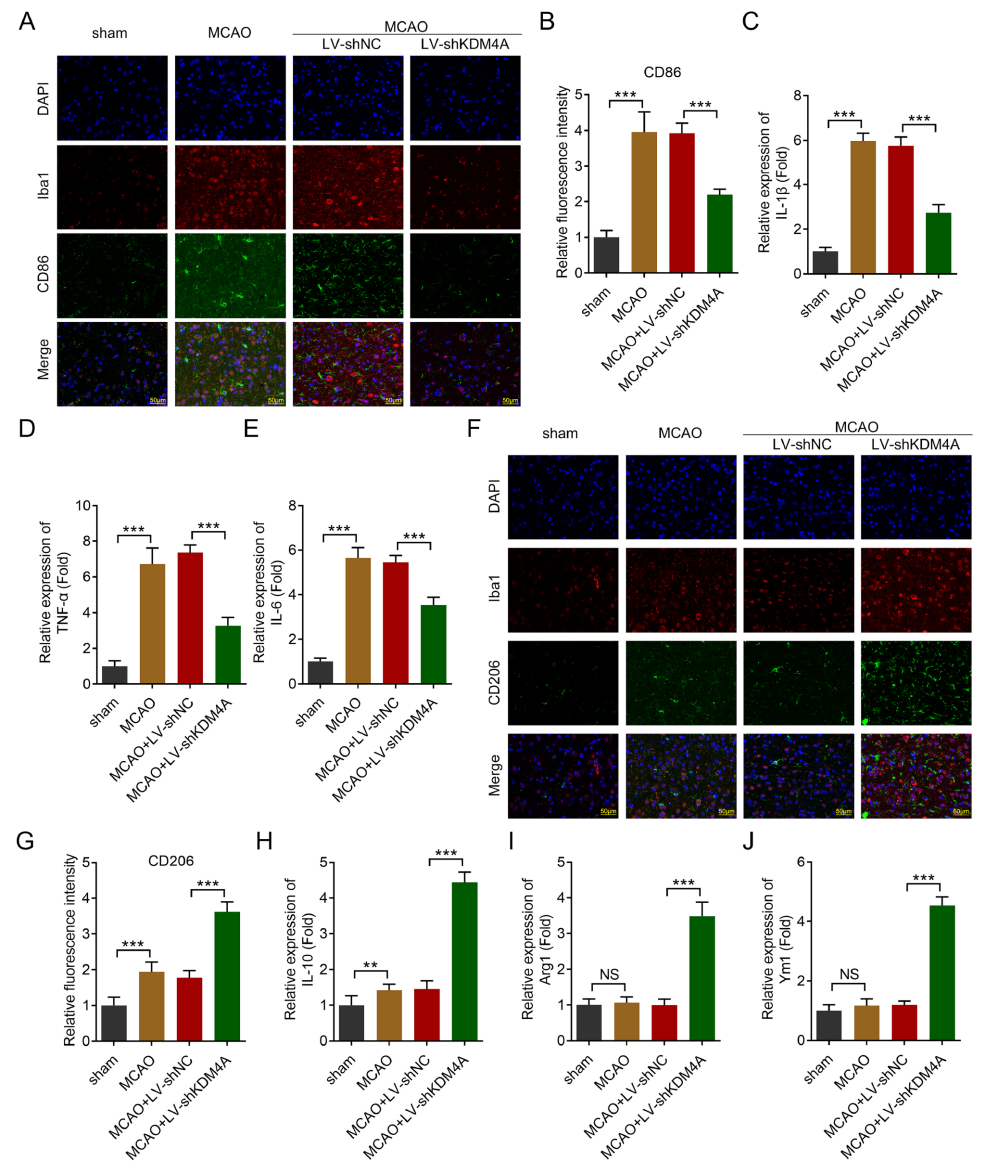
Figure 4 KDM4A deficiency inhibits M1 microglia polarization (A,B) CD86 expression in the brain tissues of the MCAO-induced IS model rats was determined via immunofluorescence assay. Scale bar: 50 μm. n = 6. (C–E) The mRNA expression of IL-1β (C), TNF-α (D), and IL-6 (E) in the brain tissues of the MCAO-induced IS model rats was detected via RT-qPCR. n = 6. (F,G) CD206 expression in the brain tissues of the MCAO-induced IS model rats was determined via an immunofluorescence assay. Scale bar: 50 μm. n = 6. (H‒J) The mRNA expression levels of IL-10 (H), Arg1 (I), and Ym1 (J) in the brain tissues of the MCAO-induced IS model rats were detected via RT-qPCR. n = 6. **P < 0.01,***P < 0.001. NS, not significant.

M1 microglial polarization is the key factor that mediates the progression of IS [28]. Thus, we hypothesized that KDM4A may be involved in the pathogenesis of IS by driving M1 microglia polarization. We found that *KDM4A* knockdown significantly alleviated the effects of MCAO surgery and reduced the percentage of M1 microglia (Iba1 ^+^CD68 ^+^) ( Figure 4A,B). Moreover, *KDM4A* knockdown significantly inhibited the release of proinflammatory cytokines ( *e*. *g*., IL-1β, TNF-α, and IL-6) by M1 microglia ( Figure 4C–E). Additionally, *KDM4A* knockdown significantly promoted an increase in the number of M2 microglia (Iba1 ^+^CD206 ^+^) ( Figure 4F,G) and increased the release of IL-10, Arg1, and Ym1 ( Figure 4H–J). These results suggested that KDM4A deficiency inhibited the switch of microglia toward M1 polarization.

### *KDM4A* deficiency alleviates neuroinflammation by downregulating SPINK5

**Figure 5:**
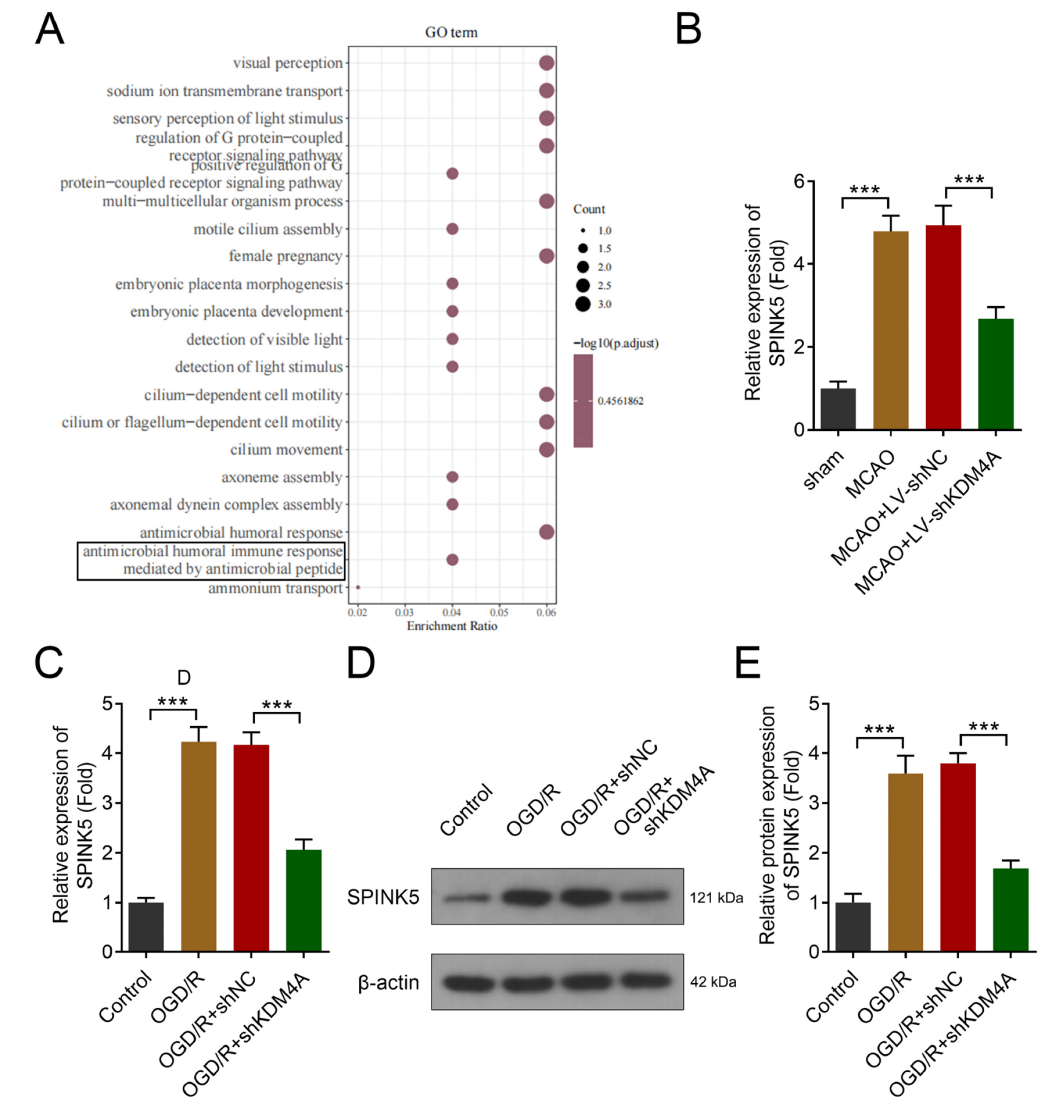
Figure 5 KDM4A deficiency alleviates neuroinflammation by downregulating SPINK5 (A) DEGs after KDM4A shRNA transfection were analyzed via GSE63812 and GO analysis. (B) SPINK5 expression in the brain tissues of the MCAO-induced IS model rats was detected via RT-qPCR. n = 6. (C) SPINK5 mRNA expression in the OGD/R-induced IS model in vitro was detected via RT-qPCR. n = 3. (D,E) SPINK5 expression in the OGD/R-induced IS model in vitro was detected by western blot analysis. n = 3. ***P < 0.001.

GSE63812 was used to analyze the DEGs after KDM4A shRNA transfection. We found that the DEGs were enriched in the antimicrobial humoral immune response mediated by antimicrobial peptide ( Figure 5A). Given the role of SPINK5 in neuroinflammation, we investigated its expression in IS models. We found that SPINK5 was significantly upregulated in the MCAO model group compared with the control group ( Figure 5B), which was reversed by *KDM4A* knockdown. Moreover, oxygen-glucose deprivation/reoxygenation (OGD/R)-mediated upregulation of SPINK5 was significantly reversed by KDM4A shRNA ( Figure 5 C–E).

### KDM4A promotes chromatin accessibility for SPINK5 by promoting H3K9 demethylation of its promoter

**Figure 6:**
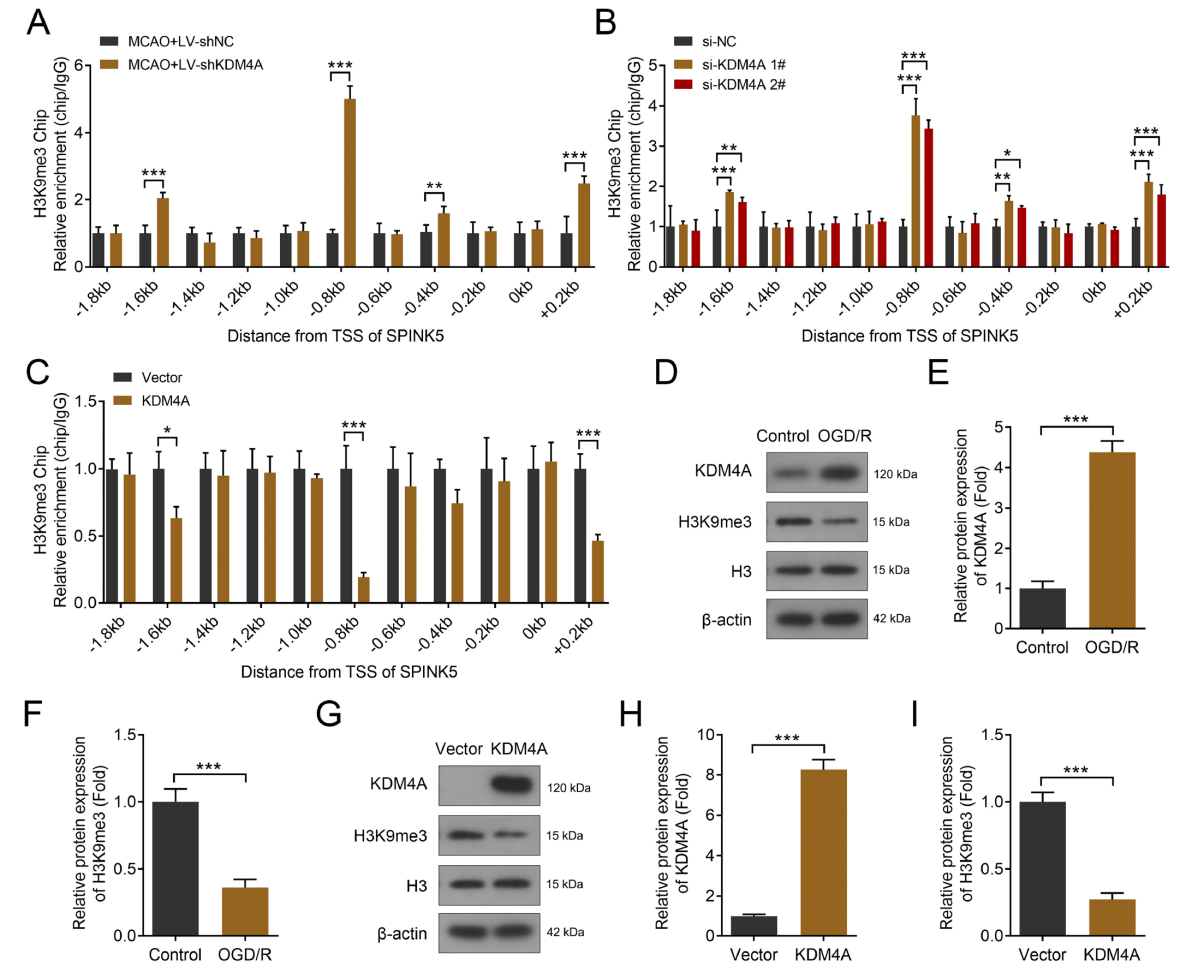
Figure 6 KDM4A promotes chromatin accessibility for *SPINK5* by promoting H3K9 demethylation of its promoter (A) H3K9me3 enrichment at the promoter of SPINK5 in the brain tissues of the MCAO-induced IS model rats was determined via ChIP assay. n = 6. (B,C) H3K9me3 enrichment in the promoter of SPINK5 in HIPA cells was determined via a ChIP assay. n = 3. (D‒F) KDM4A and H3K9me3 expression levels were determined via western blot analysis after exposure to OGD/R. n = 3. (G‒I) KDM4A and H3K9me3 expression levels were determined via western blot analysis after transfection with KDM4A overexpression plasmids. n = 3. *P < 0.05, **P < 0.01, ***P < 0.001.

KDM4A, a histone demethylase, regulates gene expression by driving the demethylation of H3K9me3 on the gene promoter of target proteins [29]. We found that H3K9me3 enrichment was significantly increased in several regions of the *SPINK5* promoter after *KDM4A* knockdown *in vivo* ( Figure 6A). This finding is consistent with the *in vitro* assay results. As shown in Figure 6B, *KDM4A* knockdown significantly increased the enrichment of H3K9me3 on the *SPINK5* promoter compared with that in the NC group. Conversely, KDM4A overexpression significantly reduced the enrichment of H3K9me3 on the *SPINK5* promoter ( Figure 6C). To further confirm these results, we determined the protein expression of H3K9me3 after exposure to OGD/R or transfection with KDM4A-overexpressing plasmids. The results revealed that OGD/R exposure significantly increased KDM4A protein expression but decreased H3K9me3 ( Figure 6D–F). Moreover, KDM4A overexpression significantly increased KDM4A protein expression while downregulating H3K9me3 protein expression ( Figure 6G–I). These results suggested that KDM4A promotes *SPINK5* expression by reducing H3K9me3 enrichment at its promoter.

### SPINK5 overexpression induces brain damage

**Figure 7:**
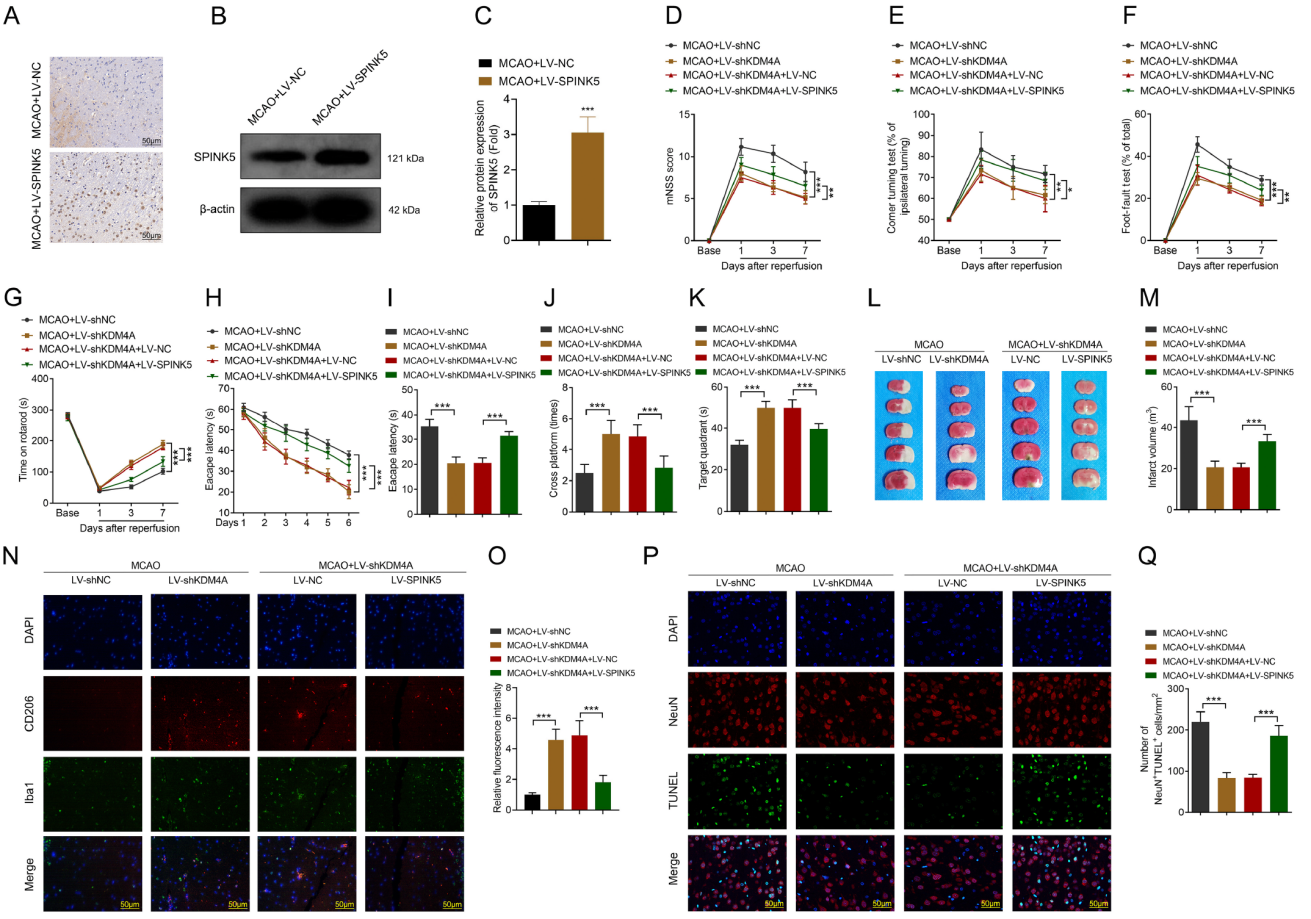
Figure 7 SPINK5 overexpression induces brain damage (A) SPINK5 expression was detected via immunohistochemistry assay. n = 6. (B,C) SPINK5 protein expression was detected via western blot analysis. n = 6. (D–G) The neurological functions and behaviors of the MCAO-induced IS model rats were determined via mNSS (D), corner-turning test (E), foot-fault test (F), and rotarod test (G). n = 6. (H‒K) Cognitive function was analyzed via Morris water maze test. n = 6. (L,M) The infarct volume of brain tissues was detected via TTC staining after MCAO surgery. n = 6. (N,O) CD206 expression in the brain tissues of the MCAO-induced IS model rats was determined via immunofluorescence assay. Scale bar: 50 μm. n = 6. (P,Q) Neuronal apoptosis in brain tissues was determined via TUNEL assay after MCAO surgery. Scale bar: 50 μm. n = 6. *P < 0.05, **P < 0.01, ***P < 0.001.

Rescue assays were conducted to further confirm the KDM4A-SPINK5 axis in IS. We found that SPINK5 expression was markedly increased after injection of LV-SPINK5 ( Figure 7A). Moreover, LV-SPINK5 significantly upregulated SPINK5 protein expression compared with that in the LV-NC group ( Figure 7B,C). After MCAO surgery, SPINK5 overexpression significantly alleviated the effects of *KDM4A* knockdown and promoted neuroinflammation, cognitive deficits, and motor impairments ( Figure 7D–G). Moreover, SPINK5 overexpression induced cognitive impairment ( Figure 7H–K). SPINK5 overexpression significantly increased the ischemic volume ( Figure 7L,M). SPINK5 overexpression significantly alleviated the effects of *KDM4A* knockdown and reduced the percentage of M2 microglia ( Figure 7N,O). Additionally, SPINK5 overexpression alleviated the effects of *KDM4A* knockdown and promoted neuronal apoptosis ( Figure 7P,Q). These results suggest that KDM4A exacerbates the progression of IS by regulating SPINK5.

## Discussion

In this study, KDM4A was found to be overexpressed in IS. KDM4A promoted the switch of microglia to the M1 phenotype and stimulated the inflammatory response. Moreover, microglial KDM4A induced neuroinflammation, cognitive deficits, and motor impairment. Conversely, KDM4A deficiency mitigated these effects, highlighting its potential as a therapeutic target for IS. Mechanistically, KDM4A upregulated SPINK5 by inhibiting H3K9 trimethylation of its promoter. SPINK5 overexpression promoted M1 microglial polarization and neuroinflammation, further supporting the role of the KDM4A-SPINK5 axis in IS pathogenesis.

Genetic alterations are key factors of IS and are modulated by transcription regulators, including histone methylation [30]. Histone modification drives microglia toward a proinflammatory phenotype and subsequent neuroinflammation, which explains how the microenvironment is reprogrammed by the epigenetic code during the pathological process of IS injury [ 31– 33]. However, the methylation status of histones affects gene transcription [34]. H3K9me3, which functions to maintain heterochromatin, inhibits transcription [35]. However, the demethylation of trimethyl groups to monomethyl groups from H3K9 may inhibit the function of H3K9me3 and promote gene transcription [36]. Das *et al*. [37] demonstrated that H3K9me3 methylation promotes recovery from acute hypoxia in the brains of male zebrafish, suggesting that H3K9me3 methylation has a protective effect on brain injury. KDM4A, a histone demethylase, promotes gene transcription by reducing H3K9me3 levels [18]. For example, KDM4A-mediated demethylation of H3K9me3 promotes neutrophil infiltration and the development of cerebral ischemia and reperfusion-induced brain injury [24]. In this study, KDM4A was found to be overexpressed in IS. KDM4A overexpression promoted M1 microglial polarization, neuroinflammation and death, resulting in brain injury and cognitive deficits. Conversely, KDM4A deficiency inhibited M1 microglia polarization and improved brain functions. Taken together, these findings suggest that KDM4A-mediated M1 microglial polarization contributes to neurodegeneration in IS.

*SPINK5*, a member of the serine protease inhibitor Kazal type (SPINK) family, encodes the lymphoid epithelial‐associated inhibitor LEKTI [38]. Increasing evidence has demonstrated that *SPINK5* functions as an antitumor gene. For example, SPINK5 downregulation promotes the growth of oral squamous cell carcinoma [39]. However, SPINK5 overexpression inhibits the metastasis of melanoma [40]. SPINK5 overexpression promotes the apoptosis of gastric cancer cells [41]. Clark *et al*. [42] reported that aberrant levels of SPINK5 are closely associated with the pathogenesis of familial essential tremor. These findings suggest that the roles of SPINK5 may vary with the type of disease. Nonetheless, the roles of SPINK5 in the pathogenesis of IS are still unknown. In this study, KDM4A-mediated demethylation of H3K9me3 increased SPINK5 expression, which promoted the inflammatory response and neuronal apoptosis. SPINK5, a negative regulator of humoral immunity, participates in regulating the inflammatory response by reprogramming the immune system [43]. In this study, KDM4A-mediated upregulation of SPINK5 promoted M1 microglial polarization, neurodegeneration and even death. However, KDM4A deficiency promoted the enrichment of anti-inflammatory cytokines (IL-10), suggesting the activation of humoral responses [44]. Therefore, we hypothesized that targeting KDM4A-humoral immunity signaling may be a potential strategy for treating IS-induced brain damage.

In conclusion, our study elucidates the role of KDM4A in IS, highlighting its ability to regulate microglial polarization and neuroinflammation via SPINK5. These findings suggest that inhibiting KDM4A may protect against ischemic brain injury by modulating microglial activity and immune responses. Future research should focus on exploring the therapeutic potential of targeting the KDM4A-SPINK5 axis in IS.
